# The Role of Dopamine in Gastric Cancer—A Systematic Review of the Pathogenesis Phenomena Developments

**DOI:** 10.3390/biomedicines12122786

**Published:** 2024-12-07

**Authors:** Radu-Cristian Cimpeanu, Dragoș Fortofoiu, Elena Sandu, Ioana-Gabriela Dragne, Mariana-Emilia Caragea, Roxana-Ioana Dumitriu-Stan, Bianca-Margareta Salmen, Lidia Boldeanu, Delia Viola Reurean-Pintilei, Cristin-Constantin Vere

**Affiliations:** 1 Doctoral School, University of Medicine and Pharmacy, 200349 Craiova, Romania; cimpeanu_r@yahoo.com (R.-C.C.); fortofoiudragos@gmail.com (D.F.); sanduelena97@gmail.com (E.S.); idragne@yahoo.com (I.-G.D.); mariana.emilia77@yahoo.com (M.-E.C.); 2 Doctoral School, “Carol Davila” University of Medicine and Pharmacy, 050474 Bucharest, Romania; roxana-ioana.dumitriu@drd.umfcd.ro; 3 Department of Microbiology, Faculty of Medicine, University of Medicine and Pharmacy of Craiova, 200349 Craiova, Romania; lidia.boldeanu@umfcv.ro; 4 Department of Medical-Surgical and Complementary Sciences, Faculty of Medicine and Biological Sciences, “Stefan cel Mare” University, 720229 Suceava, Romania; delia.pintilei@usm.ro; 5 Department of Gastroenterology, University of Medicine and Pharmacy of Craiova, 200349 Craiova, Romania; vere.cristin@gmail.com

**Keywords:** dopamine, gastric cancer, STAT-3, DARPP-32

## Abstract

Background: In the last few decades, it has been emphasized that dopamine, a well-known neurotransmitter with multiple roles in central nervous system, is also implicated in the activity of peripheral tissues and organs, more specifically influencing the gastrointestinal system (GI). Methods: We registered a protocol under the CRD42024547935 identifier in the Prospero register of systematic reviews. Furthermore, using the Population, Intervention, Comparison, Outcome, and Study Design strategy to guide our study rationale, and under the Preferred Reporting Items for Systematic reviews and Meta-Analyses recommendations, we conducted a qualitative systematic literature search based on the PubMed, Scopus, and Web of Science databases using the “gastric cancers AND dopamine” search criteria. We obtained 68 articles from PubMed, 142 articles from Scopus, and 99 articles from the Web of Science database. Results: Within gastric cancer biology, dopamine has notable effects on STAT-3 and DARPP-32. STAT-3, a transcription factor involved in cellular proliferation and invasion, plays a significant role in cancer progression. Conclusions: Understanding the roles of dopamine in cancer, beyond aspects such as cancer cell invasion, immune response modulation, or tumor growth, could guide the development of new cancer therapies by modulating its pathways, especially the DARPP-32/CXCR4/CXCL-12 complex axis, in order to improve the morbidity and mortality caused by this type of cancer.

## 1. Introduction

Dopamine (3-hydroxytyramine) is a member of the catecholamine family, being secreted by neurons that have a classic known origin in the substantia nigra. It is an essential neurotransmitter because it plays an important role in many physiological phenomena, including motor control, motivation, emotions, and cognitive functions [1,2]. Until recently, it was significantly associated with central nervous system (CNS) activity, while in the last few decades, it has been emphasized that dopamine presents other functions in influencing the activities of peripheral organs and tissues, including the gastrointestinal (GI) system, and is considered to be implied in the absorption and decarboxylation of cellular processes [3]. Regarding its roles in pathology, dopamine is linked to diverse degenerative diseases and disorders and also to tumorigenesis, as noted in the case of gastric cancer [4].

Gastric cancer currently represents one of the most common malignancies, with significant morbidity and mortality. This fact strongly demonstrates the need to identify the factors that influence its progression, especially biochemical regulators such as dopamine, which is crucial for the research and development of new therapeutic strategies, in order to minimize patient disability [5].

Regarding GI activity, the production of dopamine is realized by non-neuronal cells such as immune cells and gastric mucosal cells [6,7]. It is responsible for gastric acid secretion regulation and also for the functions of stomach lining integrity [6]. Additionally, it presents anti-inflammatory properties, balancing the immune response in the digestive system [6,8]. Dopamine inhibits the release of pro-inflammatory cytokines like interleukin-2 and interleukin-1B, which are often linked to gastric diseases, including gastric cancer [4].

Dopamine has additional function in influencing the blood flow in the GI tract, ensuring adequate oxygen and nutrient uptake of the stomach. This emphasizes its importance in maintaining the integrity and function of the stomach, while any disturbances in this system regulation might be responsible for the development of gastric diseases, including gastric cancer [6,9,10].

Dopamine’s action is based on its binding to dopamine receptors and triggering intracellular signaling chains. The expression of dopaminergic receptors (DRs) is widely distributed in different organs, such as the brain, retina, heart, coronary arteries, sympathetic ganglia, and GI tract. The DRs are members of the seven transmembrane G protein-coupled receptor families, which are classified into D1-like receptors and D2-like receptors. The first type, D1-like receptors, includes D1 and D5 receptors, which are involved in the activation of adenylyl cyclase activity, while the second type, D2-like receptors, represented by D2, D3, and D4 receptors, has antagonistic effects on D1-like receptors [11,12]. Moreover, through D2-like receptors, dopamine induces Vascular Endothelial Growth Factor (VEGF) inhibition, which is associated with poor prognoses in angiogenesis processes [13,14]. D2 has been found to inhibit insulin growth factor 1 (IGF-I)-induced gastric cancer cell growth. For D2-like receptors, polymorphisms within the receptor gene have been shown to be associated with colorectal cancer risk, and the expression of D2-like receptor is also inversely correlated with the prognosis of patients with gastric cancer [14,15]. In a study that evaluated 84 pairs of tumor and adjacent non-tumor tissues, immunochemical analysis was used to detect the expression levels of D2-like receptor in the tissues, and D2-like receptor was expressed at a higher level in tumors compared with adjacent matched non-tumor tissues [14]. Patients with higher expression levels of D2 DR had lower survival durations. The expression of D2-like DR was negatively associated with the survival durations of patients with gastric cancer [14].

Tumoral angiogenesis is an essential step in solid tumor growth and progression. In this direction, in the literature, it is stipulated that VEGF-mediated angiogenesis is strongly and specifically inhibited by dopamine. In some types of cancers, dopamine has been shown to inhibit angiogenesis [13]. By limiting angiogenesis, dopamine can decelerate tumor growth; the discovery of the exact mechanisms could be an important step forward in treating gastric cancer.

Dopamine is also involved in the immune system’s response with regard to the tumors’ activity. Altering the activity of the T cells immune and macrophages, it might be responsible for immune system suppression that due to the recognition and action of targeted tumoral cells [15,16]. On the one hand, dopamine inhibits cancer growth; on the other hand, it could increase the tumor activity, which could complicate the impact of cancer progression.

With regard to gastric cancer, dopamine is also a major factor involved in inflammation regulation, influencing chronic inflammation and inducing oxidative stress, DNA mutations, and anti-inflammatory effects while reducing the production of tumor necrosis factor α and interleukin 6 (IL-6) [17].

The aim of this systematic review is to assess the most recent data regarding the link between dopamine activity and gastric cancer in human patients.

## 2. Materials and Methods

We registered a systematic review protocol under the CRD42024547935 number in Prospero, which followed the recommendations of Preferred Reporting Items for Systematic Reviews and Meta-Analyses. Furthermore, using the Population, Intervention, Comparison, Outcome, and Study Design, we developed the strategy that guided our study rationale.

### 2.1. Research Question and Search Strategy

We conducted a qualitative systematic search of the literature, based on PubMed, Scopus and Web of Science databases, using the “gastric cancers AND dopamine” search criteria.

We obtained 68 articles in PubMed, 142 articles in Scopus, and 99 articles in the Web of Science database.

### 2.2. Inclusion Criteria

The inclusion criteria were original full-text articles, randomized control trials, and clinical trials published in English from 1 January 2014 up to 31 May 2024 that refer to the role of dopamine in the pathogenesis process of gastric cancer in the human population.

### 2.3. Exclusion Criteria

The exclusion criteria were case reports, reviews, meta-analyses, letters to the editors, duplicates, articles that lacked originality, articles published in languages other than English, those on non-human populations or only on cell cultures or cell lines, and those that evaluated the diagnosis and therapeutic processes.

### 2.4. Studies Selection

Studies that met the eligibility criteria (1) included human patients with gastric cancer and dopamine assessment; (2) evaluated the relationship between dopamine level and gastric cancer; (3) provided sufficient information such as 95% confidence intervals or, at least, p-value. Studies were excluded if they (1) were redundant publications; (2) provided insufficient or incomplete data; (3) were realized only on cell lines or on cell cultures; or (4) were case reports, letters to the editor, meeting abstracts, expert opinions, or reviews.

### 2.5. Data Extractions

Two researchers extracted the studies’ titles and abstracts, screened them for relevance for the present study theme, searched for the presence of at least one analysis of a human population with gastric cancer, and selected the relevant ones by performing cross-screening. If any disagreements occurred in the selection process, these were settled by a third reviewer.

We also performed a manual search of the databases to identify other potentially useful articles missed by our search strategy and identified an article.

### 2.6. Risk of Bias Assessment

Bias assessment was carried out using the Newcastle–Ottawa scale (NOS) [18].

### 2.7. Strategy of Data Synthesis

After the selection process took place and the articles were evaluated by the reviewers, only 4 articles were included. Furthermore, a narrative synthesis of the findings from the studies centered around the dopamine level in human populations with gastric cancer and its relationship with the disease was made. Because the studies were expected to be heterogeneous in terms of study design and quality and the screening methods, interventions, and outcomes described, a narrative synthesis was performed using text and tables in order to provide a descriptive summary and explanation of the study characteristics and findings. The current article is referring to about the last 10 years of developments in understanding dopamine’s involvement in the pathogenic mechanisms in human populations with gastric cancer, reviewing recently discovered genetic notions about this topic. This article also excludes articles that report already-known information on cell cultures or cell lines and pathology features in histologically typical aspects.

## 3. Results

**Figure 1:**
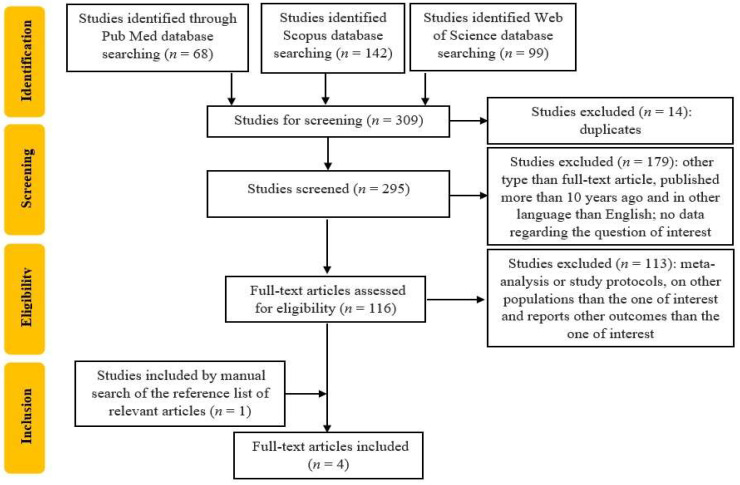
Flowchart of the study selection process.

The entire selection process led to incorporation of four studies, which were published between 2014 and 2024. The process is summarized in Figure 1.

NOS was used for risk of bias assessment, and it was conducted by two reviewers. They independently assessed the quality of the studies using a star rating system that evaluated the selection, comparability, and outcome criteria of the articles. The results are shown in Table 1.

From the four included studies, we collected data regarding the evaluation method of the samples, type of samples, specifically evaluated parameters, and their outcomes with their measurements. They are summarized in Table 2.

## 4. Discussion

Gastric cancer is in the top 10 malignancies worldwide. Moreover, it is recognized as being the fifth cancer in terms of its incidence and the fourth in mortality rate. According to the International Agency for Research on Cancer, more than one million new cases of gastric cancer were diagnosed in 2020, and, unfortunately, it led to the death of 769,000 patients [23].

Dopamine is a neurotransmitter that is poorly investigated considering its importance, especially in cancer gastric pathophysiology. Dopamine has been evaluated in in vitro assays, cancer cell lines, three-dimensional gastric gland organoid cultures, mouse models, and human tissue samples, and the majority of the published studies in the literature refer to its therapeutically discovered roles rather than its pathophysiology [23,24,25,26,27,28,29,30].

Regarding the implication of dopamine in the pathogenesis of gastric cancer, we identified that the most used methods for the evaluation of the sample tissues were immunohistochemistry (IHC) and Polymerase Chain Reaction techniques (qRT-PCR). The analysis of human gastric cancer tissue microarrays shows high levels of dopamine- and cAMP-regulated phosphoprotein Mr 32000 (DARPP-32) and positive immunostaining for nuclear signal transducer and activator of transcription 3 (STAT3) in cancer tissues as compared to non-cancer, histologically normal tissues.

### 4.1. The Direct Relation Between STAT-3 and DARPP-32

STAT-3 is a type of transcription factor that is located on the 17q21 chromosome, and it is recognized as a DNA-binding factor. STAT-3 presents a selective capacity for binding to the IL-6, being a responsive element which is directed to hepatocyte stimulation, as well as being involved in epidermal growth factor activity [23,31]. Also, STAT-3 is identified as being involved in carcinogenesis, presenting a role in cellular proliferation and cellular invasion, and having the capacity for angiogenesis mediation and metastasis [23,31,32].

DARPP-32, a protein involved in tumorigenesis, demonstrated that, in its transient form, it increases STAT-3 expression in human gastric carcinoma epithelial cell line (AGS) cells, while in the endogenous form, this protein has the opposite effect in MKN-45 cells [33].

Overall, DARPP-32 overexpression has been recognized in more than 70% of cancers, while STAT-3 is phosphorylated and plays an important role in all steps of tumorigenesis [33].

### 4.2. The Correlation Between DARPP-32, t-DARPP, mRNA, and ANGPT2

*Angiopoietin-2* (ANGPT-2) is a gene for Angiopoietin 2 codification and a member of the ANGPT-TIE System. It is correlated with a poor prognosis and has an overexpression role through DARPP-32 influence, thus resulting in STAT3 activation. In addition, DARPP-32 alternatively encodes mRNA that generates an isoform protein named truncated (t-DARPP-32), which is also overexpressed in gastric adenocarcinoma [33,34,35].

### 4.3. The Correlation Between CD44E, DARPP-32, and SRp20

DARPP-32 regulates CD44E expression by modulating SRp20 in the progression of gastric cancer, shedding light on the complex mechanisms by which DARPP-32 enhances splicing activity to support tumor development. Changes in DARPP-32 expression significantly affected CD44E expression in terms of both mRNA and protein levels. Specifically, minimizing DARPP-32 was followed by a reduction in CD44E levels, highlighting DARPP-32’s influence on splicing and the inclusion of specific exons essential for cancer progression. Moreover, CD44 was reported to have its glycosylation profile affected in gastric cancer alongside other intestinal cancers [36].

When SRp20 is inhibited, CD44E levels drop substantially, establishing SRp20 as a central mediator of DARPP-32’s splicing enhancement and making SRp20 a direct mediator of DARPP-32’s activity.

Co-immunoprecipitation revealed an interaction between DARPP-32 and SRp20, where DARPP-32 was found to extend SRp20’s half-life and reduce its ubiquitination and degradation, thus stabilizing SRp20 within the cell.

In MKN-45 cells, a rescue experiment reinforced the role of the DARPP-32-SRp20 axis in CD44E regulation. The combined overexpression of DARPP-32 and SRp20 restored CD44E levels that were otherwise reduced by DARPP-32 knockdown. These findings were supported in an in vivo xenograft model, showing that tumors with reduced DARPP-32 had slower growth and lower CD44E levels, while SRp20 overexpression partially restored both growth and CD44E expression [37].

Patient sample analysis revealed that gastric tumors exhibited higher levels of DARPP-32, CD44E, and SRp20 mRNAs compared to normal tissues, with significant correlations among these factors. This underscores their clinical relevance in gastric cancer. DARPP-32 enhances CD44E expression via SRp20 stabilization, increasing splicing activity and contributing to gastric cancer development. The DARPP-32-SRp20-CD44E pathway could be considered a potential therapeutic target in gastric cancer treatment.

### 4.4. The Correlation Between DARPP-32, CXCR-4, and CXCL-12

DARPP-32, known for its role in tumor progression, was observed to significantly increase invasive activity in gastric cancer cells. When DARPP-32 was knocked down in MKN-45 cells, a noticeable reduction in invasive behavior was observed, underscoring DARPP-32’s influence on cell invasion.

In CXC motif chemokine 12 (CXCL-12)-mediated invasion assays, DARPP-32 overexpression was shown to enhance cancer cell invasion in response to CXCL-12, suggesting that DARPP-32 is actively involved in chemokine-driven metastasis. Additional experiments using an endothelial cell invasion model further supported DARPP-32’s function in boosting invasive activity, confirming its role as a key promoter of invasion.

One critical pathway identified in this process involves DARPP-32’s impact on C-X-C chemokine receptor type 4 (CXCR4) and membrane type 1 matrix metalloproteinase (MT1-MMP) expression, which subsequently activates MMP-2, an enzyme essential for extracellular matrix breakdown during invasion. DARPP-32 overexpression resulted in a marked increase in MMP-2 activity, whereas DARPP-32 knockdown diminished it, indicating a regulatory mechanism dependent on DARPP-32. Although changes in CXCR4 mRNA were not observed with DARPP-32 manipulation, protein levels of CXCR4 and MT1-MMP rose with DARPP-32 overexpression, suggesting post-translational control. Studies revealed that DARPP-32 and CXCR4 form a protein complex that stabilizes CXCR4 by reducing its ubiquitination and prolonging its half-life, thereby enhancing CXCR4’s stability on the cell membrane [22].

Additionally, DARPP-32’s interaction with CXCR4 influences CXCR4 localization in response to CXCL-12.

Also, CXCR4 and DARPP-32 reduce the invasive activity in DARPP-32-expressing cells. Blocking CXCR4 led to decreased MT1-MMP expression, further linking CXCR4 activity with MT1-MMP regulation. These findings collectively underscore the central role of CXCR4 in the DARPP-32-enhanced invasion pathway in gastric cancer cells.

In gastric tumor samples, elevated levels of DARPP-32, CXCR4, and CXCL-12 were noted compared to normal tissues, with statistical analysis confirming positive correlations between DARPP-32 and both CXCR4 and CXCL-12. This consistent expression pattern among these invasion-associated proteins in gastric cancer highlights the significance of the DARPP-32/CXCR4/CXCL-12 axis in promoting invasion. This knowledge suggests that targeting this pathway could serve as a therapeutic strategy for managing metastatic progression in gastric cancer.

### 4.5. The Expression of DRD5 mRNA

Furthermore, regarding the last 10 years of publications about this topic, in gastric cancer, dopamine receptor D5 (DRD5) activation is able to suppress growth tumor if there are specific substances that target this receptor, inducing an autophagic cell death process.

Another association was established between D2R and IGF-1. IGF-1 plays a critical role in the stimulation of gastric cancer cell proliferation, angiogenesis, survival, and resistance to apoptosis [29]. There is significant higher expression of phosphorylated IGF-IR in gastric cancer cells compared to non-tumor cells. D2R inhibits IGF-IR-induced tumor cell proliferation, and studies have shown that D2R agonists may be an effective choice for gastric cancer treatment.

### 4.6. Integrating the Existing Knowledge for Future Directions

Dopamine’s effects are mediated through two receptor types: D1-like (D1, D5), which activate adenylyl cyclase, and D2-like (D2, D3, D4), which inhibit it. After binding to D2-like receptors, dopamine also induces DRD2 and VEGF.

Concerning gastric cancer biology, dopamine has notable effects on STAT-3 and DARPP-32. STAT-3, a transcription factor involved in cellular proliferation and invasion, plays a significant role in cancer progression. Dopamine’s effect on DARPP-32—a tumor-promoting protein—has been shown to increase STAT-3 expression in AGS cells, while in MKN-45 cells, endogenous DARPP-32 actually suppresses STAT-3. This dual regulatory action is crucial, as DARPP-32 overexpression is described in over 70% of cancers, while phosphorylated STAT-3 is integrally involved in tumor development.

DARPP-32 also shows a significant connection to t-DARPP, mRNA, and *ANGPT-2*. *ANGPT-2*, a gene involved in angiogenesis, is linked to poor survival and is upregulated by DARPP-32 through STAT-3 activation. DARPP-32, along with its truncated form, t-DARPP, influences gene expression and contributes to cancer development, highlighting its role in alternative mRNA splicing.

In regulating CD44E, DARPP-32 acts by stabilizing SRp20, a critical splicing factor. SRp20’s stabilization allows DARPP-32 to influence CD44E splicing, an essential form for tumor progression. Reductions in DARPP-32 expression lead to lower CD44E levels, while knockdowns of SRp20 similarly decrease CD44E, showing SRp20 as a mediator in DARPP-32’s splicing activity. In vivo studies and patient sample analyses confirm elevated levels of DARPP-32, CD44E, and SRp20 in gastric tumors, suggesting that the DARPP-32-SRp20-CD44E pathway could be a viable therapeutic target.

Additionally, DARPP-32 significantly impacts CXCR4 and CXCL-12 expression, critical for invasive behavior in gastric cancer. DARPP-32 overexpression boosts CXCL-12-mediated cell invasion, showing its active role in chemokine-driven metastasis. Through this pathway, DARPP-32 upregulates CXCR4 and MT1-MMP, enhancing MMP-2 activity, an enzyme necessary for extracellular matrix degradation and invasion. Although CXCR4 mRNA remains unaffected, DARPP-32 upregulates CXCR4 protein levels, indicating post-translational regulation. DARPP-32 also stabilizes CXCR4 by reducing its ubiquitination, which prolongs its activity and supports metastatic potential.

**Figure 2:**
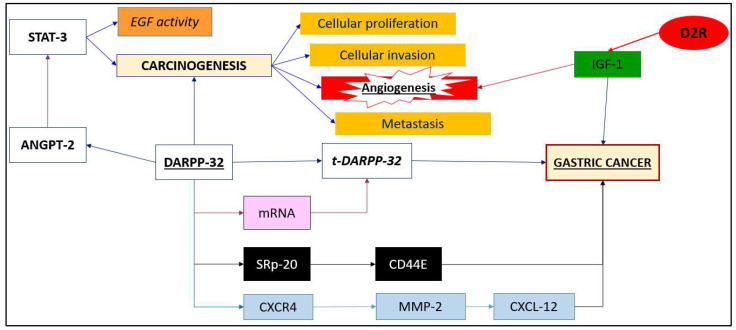
The summarized correlation between molecular mechanisms previously described of dopamine in gastric cancer: dopamine receptor 2 (noted in red circle), is the initiator of this processes and his effect is modulated by IGF-1 (noted in green rectangular form). There are different pathways in gastric cancer development (mentioned with yellow), the main effect that promotes tumorigenesis being angiogenesis (noted in red rectangular form), with purple, black, and blue being emphasized different dopamine pathways that together form the DARPP-32/CXCR4/CXCL-12 axis that generate gastric cancer appearance through dopamine involvement.

Inhibiting CXCR4 through AMD3100 or siRNA significantly reduces DARPP-32-induced invasion. CXCR4 inhibition also lowers MT1-MMP expression, underscoring CXCR4’s central role in the DARPP-32-driven invasion pathway. Studies of gastric tumor samples demonstrate increased DARPP-32, CXCR4, and CXCL-12 compared to normal tissues, with correlations between these proteins suggesting that targeting the DARPP-32/CXCR4/CXCL-12 pathway could effectively manage metastasis in gastric cancer, as shown in Figure 2.

### 4.7. Importance of Pathogenic Knowledge in Future Treatment Field

Gastric cancer remains a debilitating pathology, as its evolution and main treatment, represented by surgery or endoscopic resection, lead to nutritional deficiencies, along with subsequent consequences such as reinterventions or strictures. Despite the knowledge of the pathogenic and gene development mechanisms regarding dopamine’s involvement in gastric cancer, the currently represented direction of treatment is perfectible and could be more specific in targeted drug therapy [38,39,40,41,42,43,44,45].

Moreover, in some cases in which resection invasive therapy was performed, some of the excised tumors presented recurrence and metastatic processes, probably due a production by a central nervous system lesion mediated by nerve fibers releasing unbalanced dopamine and a persistent disturbance of neurotransmitters, as well as full hyperplasia or dysplasia features of enterochromaffin-like cells [46,47,48,49].

Most of the studies in the last decade have acknowledged the effect pathological analyses focused on cell cultures or cell lines on murine model or human tissues, emphasizing the main features and, for some of them, the peculiarities, but most of them have not isolated the gene involved in neuroendocrine tumorigenesis. The main issue is still identifying the variability and completing the description of gene expression in tumor tissue. In this direction, further research could improve the chances of developing more specific, new, and efficient targeted drug therapies, with the aim to promote possible curative options for patients with gastric cancer [24,28,50,51].

A limitation of the present study is the small number of included studies, which was due to our proposed aim of evaluating only human patients and not cell cultures or cell lines, as well as due to the protocol for generating a systematic review. The main strength of the present study is represented by the comprehensive approach to the pathophysiologic mechanisms that are involved in gastric cancer, especially regarding dopamine activity and the multiple pathways involved in its appearance.

## 5. Conclusions

In summary, dopamine’s influence reaches beyond its well-known role in the brain, affecting peripheral systems like the GI tract and exhibiting important activity in gastric cancer progression. Through complex interactions with molecules such as DARPP-32, STAT-3, CXCR4, and CXCL-12, dopamine contributes to cancer cell invasion, immune response modulation, and tumor growth by stabilizing key proteins involved in angiogenesis and metastasis. These findings highlight dopamine’s potential as a therapeutic target, including DRD5 expression, suggesting that treatments aimed at modulating dopamine’s pathways could offer promising strategies for gastric cancer management. With a deeper understanding of these mechanisms, the DARPP-32/CXCR4/CXCL-12 complex axis should be considered a type of targeted therapy to inhibit tumor progression and improve outcomes in patients with gastric cancer.

## Figures and Tables

**Table 1:** Newcastle–Ottawa scale analysis of the included articles.

Author (Reference)	Selection				Comparability	Outcome			Total Score	Quality
	Representativeness of the Exposed Cohort	Selection of the Non-Exposed Cohort	Ascertainment of Exposure	Demonstration That Outcome of Interest Was Not Present at Start of Study	Comparability of Cohorts Based on the Design or Analysis	Assessment of Outcome	Was Follow-Up Long Enough for Outcomes to Occur	Adequacy of Follow-up of Cohorts		
Zhu et al. [19], 2019	*	*	*	*	*	*	*	-	7	Good
Chen et al. [20], 2016	-	*	*	*	*	*	*	-	6	Good
Zhu et al. [21], 2016	*	*	*	*	-	*	*	-	6	Good
Zhu et al. [22], 2013	*	*	*	*	*	*	*	-	7	Good

“*” indicates that the article meets the criteria mentioned above; “-” indicates that the article does not meet the abovementioned criteria.

**Table 2:** Evidence of discriminatory elements found in the relevant articles.

Author	Evaluation Method	Sample	Parameters	Outcome of the Parameter	Measure of Outcome	*p* Value
Zhu et al. [19], 2019	IHC	Human gastric cancer tissue (adenocarcinomas) as compared to normal tissue	*p*–STAT3 (Y705)	Stronger immunostaining	NR	*p* < 0.01
				CES increase in gastric tumors	NR	*p* < 0.01
				Low expression associated with better survival	HR = 1.18 (1, 1.4)	*p* = 0.05
			DARPP-32	Stronger immunostaining	NR	*p* < 0.01
				CES increase in gastric tumors	NR	*p* < 0.01
				Low expression associated with better survival	HR = 1.37 (1.1, 1.7)	*p* = 0.04
Chen et al. [20], 2016	IHC	Human gastric cancer tissue (adenocarcinomas) as compared to normal tissue	DARPP-32, t-DARPP, and ANGPT2	Higher expression in gastric cancer samples	NR	*p* < 0.05
	qRT-PCR		mRNA expression levels between ANGPT2 and DARPP-32	Positive association	r2 = 0.4	*p* < 0.0001
			mRNA expression levels between t-DARPP and DARPP-32	Positive association	r2 = 0.4	*p* < 0.0001
			ANGPT2	Low levels	NR	NR
			DARPP-32	High levels and more blood vessels	NR	NR
Zhu et al. [21], 2016	qRT-PCR	Human gastric cancer tissue (adenocarcinomas) as compared to normal tissue	mRNA levels of DARPP-32	Higher expression level	65.4%	*p* < 0.001
			mRNA levels of CD44E	Higher expression level	69.2%	*p* < 0.001
			mRNA levels of SRp20	Higher expression level	76.9%	*p* < 0.001
			CD44E and DARPP-32	Positive correlation and higher expression in gastric tissue	r2 = 0.45	*p* < 0.001
			SRp20 and DARPP-32	Positive correlation and higher expression in gastric tissue	r2 = 0.70	*p* < 0.001
Zhu et al. [22], 2013	qRT-PCR	Human gastric cancer tissue (adenocarcinomas) as compared to normal tissue	DARPP-32, CXCR4 and CXCL-12	High levels in tumors as compared to adjacent normal tissues		*p* < 0.01
			CXCR4 and DARPP-32	Positive association	r2 = 0.636	NR
			CXCL-12 and DARPP-32	Positive association	r2 = 0.397	NR

IHC—immunohistochemistry; qRT-PCR—Real-Time Quantitative Reverse Transcription PCR; NR—not reported; CES—composite expression score; HR—hazard ratio; DARPP-32—dopamine- and cAMP-regulated phosphoprotein; CXCR4—C-X-C chemokine receptor type 4; CXCL-12—CXC motif chemokine 12.
